# Lipid-dependent regulation of Kv10.1 in cancer: implications of membrane remodeling for channel function and pharmacology

**DOI:** 10.3389/fphar.2026.1905919

**Published:** 2026-09-03

**Authors:** Nguyen Van Phuong, Anna Stary-Weinzinger

**Affiliations:** 1 Department of Pharmaceutical Sciences, Division of Pharmacology and Toxicology, University of Vienna, Vienna, Austria; 2 Vienna Doctoral School of Pharmaceutical, Nutritional and Sport Sciences (PhaNuSpo), University of Vienna, Vienna, Austria

**Keywords:** anticancer drug design, cholesterol modulation, Kv10.1 regulation, lipid-protein interactions, membrane lipid remodeling

## Abstract

The voltage-gated potassium channel Kv10.1 (Eag1), which is abnormally overexpressed across a wide range of tumor types, is a promising therapeutic target for cancer treatment. However, the clinical development of Kv10.1 inhibitors is currently constrained by severe off-target cardiotoxicity resulting from unintended inhibition of the structurally homologous Kv11.1 (hERG) channel. In this review, we examined cancer-associated membrane lipid remodelling and its implications for the lipid-dependent regulation of both channels. Emerging evidence suggests that membrane lipids play a critical role in regulating voltage-gated potassium channels and that, despite their substantial structural similarity, Kv10.1 and Kv11.1 are modulated by distinct lipid-dependent mechanisms. In particular, we highlighted their differential responses to key membrane constituents, including phosphatidylinositol 4,5-bisphosphate (PIP2), cholesterol, and polyunsaturated fatty acids (PUFAs). These observations suggest that the surrounding membrane environment may represent an additional and largely underexplored factor influencing channel function. We further discuss how disease-associated lipid remodeling in cancer may contribute to altered Kv10.1 activity and highlight the possibility that such changes could influence pharmacological responses. Although the underlying mechanisms remain incompletely understood, this perspective points to an underexplored dimension in potassium channel biology that may inform future therapeutic strategies.

## 1 Introduction

Cancer is one of the most significant global health challenges, with approximately 20 million incident cases and 9.7 million deaths each year (Liu and Zheng, 2024). This burden is expected to increase in the coming decades, driven by population aging and rising exposure to other cancer-related risk factors (Liu and Zheng, 2024; Filho et al., 2025). According to the World Health Organization (WHO), the demand for oncology services, including screening, diagnosis, and treatment is anticipated to grow substantially, particularly in low- and middle-income countries (WHO, 2024). Although current therapeutic approaches, such as radiotherapy, chemotherapy, targeted therapy, and immunotherapy, have markedly improved survival outcomes for many cancer types, significant limitations remain, such as drug resistance, treatment-related toxicity, and the high financial cost associated with advanced therapies (Xia et al., 2024). In addition to identifying new molecular targets, there is increasing recognition that the cellular context in which these targets operate, including the membrane environment, may influence their functional and pharmacological properties, offering new opportunities to develop more effective cancer treatments (Zalba and Ten Hagen, 2017).

Kv10.1 (also known as Eag1) is a voltage-gated potassium channel belonging to the *ether-à-go-go* family and is encoded by the *KCNH1* gene (Luis et al., 2022). The structure of Kv10.1 is a tetramer composed of four subunits, which is a defining feature of voltage-gated potassium channels (Luis et al., 2022). Under physiological conditions, Kv10.1 expression is predominantly restricted to specific regions of the central nervous system, while expression in peripheral (non-CNS) tissues is either negligible or undetectable (Zheng and Chen, 2024). In contrast, abnormal overexpression of Kv10.1 has been documented in a broad spectrum of human malignancies, i.e., up to 70% of clinical tumor samples and cancer cell lines (Hemmerlein et al., 2006; Ma et al., 2024a). Notably, Kv10.1 has been detected in 85.3% of brain metastases and 77.5% of glioblastoma cases (Martínez et al., 2015). These observations suggest that Kv10.1 could be a promising therapeutic target for oncological drug development. Supporting this hypothesis, preclinical studies have demonstrated that pharmacological inhibition of Kv10.1 activity using small molecules (e.g., astemizole) or suppressing Kv10.1 expression by siRNA significantly reduces cancer cell proliferation and migration as well as suppresses tumor growth *in vivo* (Weber et al., 2006; Downie et al., 2008; Bernal-Ramos et al., 2017).

However, a significant drawback in the development of Kv10.1-targeted antineoplastic agents is the difficulty in achieving high selectivity over other voltage-gated potassium channels, particularly Kv11.1 (also known as hERG) (Toplak et al., 2021; Toplak et al., 2022). Kv11.1 plays an important role in the repolarization of cardiac myocytes. Unintended inhibition of this channel can lead to serious cardiovascular problems, such as long QT syndrome, arrhythmias, and potentially life-threatening cardiac events (Kalyaanamoorthy and Barakat, 2018; Sanguinetti, 2023). Recently, the U.S. FDA required all clinical drug candidates to be evaluated for their potential to prolong cardiac action potentials through hERG channel blockade (Darpo et al., 2006; Sanches et al., 2024; Delpy et al., 2025). However, due to the high similarity in structure between Kv10.1 and Kv11.1, especially within the pore domain, most known Kv10.1 inhibitors, such as astemizole and imipramine, exhibit significant cross-reactivity and concurrently block Kv11.1, thereby limiting their therapeutic application in clinical use (Luis et al., 2022; Toplak et al., 2022). This highlights the critical need for the development of drugs that can selectively target Kv10.1 while sparing Kv11.1. Recent studies have indicated that although the pore domains of these channels are highly conserved, there are differences in other regions, such as the voltage-sensor domains and the intracellular N- and C-terminus, which may provide opportunities for designing ligands capable of binding selectively to allosteric pockets unique to Kv10.1 (Gubič et al., 2022). However, progress in exploiting these distinctions for therapeutic development remains limited. Importantly, these approaches have largely focused on intrinsic structural differences between the channels, while the potential contribution of the surrounding membrane environment has received comparatively little attention.

Voltage-gated potassium channels, including Kv10.1 and Kv11.1, are transmembrane proteins embedded within a complex and heterogeneous lipid environment. Numerous studies have shown that membrane lipids play an important role in modulating the function of these channels (Boland and Drzewiecki, 2008; Zheng et al., 2011; Poveda et al., 2014; Poveda et al., 2017; Cordero-Morales and Vásquez, 2018). These lipids, such as cholesterol, phosphoinositides (e.g., PIP_2_), and sphingolipids, could influence ion channel dynamics via three mechanisms: specific lipid-protein interactions; alterations in membrane physicochemical properties (such as thickness and lateral pressure); and the facilitation of channel organization within membrane signaling complexes (Rosenhouse-Dantsker et al., 2012). Concurrently, recent literature highlights the phenomenon of cancer-associated membrane lipid remodelling. This process causes significant remodelling in the molar ratios of phospholipids, cholesterol, and glycosphingolipids, as well as variations in fatty acid chain saturation and lipid raft composition, all of which fundamentally shift the physicochemical landscape of the cell membrane (Szlasa et al., 2020). In this context, disease-associated lipid remodeling may represent an additional and largely underexplored factor shaping channel behavior. For example, Shigetoshi Oiki *et al.* showed that 16:0–18:1-phospho-L-serine (POPS) in the inner leaflet selectively accelerated activation kinetics of KvAP, suggesting a limited modulatory effect on the voltage-sensor domain (VSD). This specific kinetic effect was not observed when POPS was present in the outer leaflet, a condition that is frequently associated with cancer cells due to the externalization of phosphatidylserine (Maki et al., 2025). In another study, Chase M. Fiore et al. demonstrated that the lipid composition of the plasma membrane can significantly influence the ability of certain drugs to inhibit Kv11.1 channel activity. In particular, alterations in membrane cholesterol levels were shown to modify the sensitivity of hERG channels to blockade by specific compounds, highlighting potentially important clinical implications for drug efficacy and cardiotoxicity (Fiore et al., 2025). Despite this, the role of disease-associated lipid remodeling in shaping ion channel function has received comparatively little attention.

Given that Kv10.1 is preferentially overexpressed in neoplastic cells while remaining negligible in healthy peripheral tissues, the distinct lipid composition of the cancer cell membrane may influence Kv10.1 function in a manner that differs from other potassium channels, particularly Kv11.1. Therefore, in this review, we will examine how membrane lipid composition and remodeling may contribute to the regulation of Kv10.1 and discuss how this membrane context could influence both channel function and, potentially, pharmacological responses.

## 2 Lipidomic remodelling in cancer membranes

### 2.1 Lipid composition of the plasma membrane

The mammalian plasma membrane (PM) exhibits pronounced transbilayer asymmetry in both lipid composition and mechanical properties, a feature actively maintained by ATP-dependent lipid transporters such as flippases, floppases, and scramblases (Fan et al., 2018; Sakuragi and Nagata, 2023). This asymmetry is physiologically critical. For example, the externalization of phosphatidylserine (PS) to the outer leaflet serves as a canonical hallmark of apoptosis (Lee et al., 2013).

In the plasma membrane, the exoplasmic (outer) leaflet is enriched in neutral phospholipids such as sphingomyelin (SM) and phosphatidylcholine (PC) (Ingólfsson et al., 2014). This lipid composition contributes to an electrically neutral surface, increased bilayer thickness, and higher lipid order, collectively enhancing mechanical stability against the extracellular environment (Van Meer et al., 2008). Additionally, the outer leaflet contains glycosphingolipids and gangliosides (e.g., GM1, GM3), which play essential roles in cell-cell recognition, adhesion, and the formation of protein scaffolds that regulate diverse signaling pathways (Todeschini and Hakomori, 2008).

In contrast, the cytosolic (inner) leaflet is abundant in phosphatidylethanolamine (PE), phosphatidylserine (PS), phosphatidic acid (PA), and phosphatidylinositol (PI), along with its phosphorylated derivatives, the phosphatidylinositol phosphates (PIPs) (Ingólfsson et al., 2014). This distinct lipid composition provides the inner leaflet with unique functional properties. PE contributes to negative membrane curvature, thereby facilitating dynamic remodelling processes such as membrane budding, fission, and fusion (Chernomordik and Kozlov, 2003; Holthuis, 2004). Meanwhile, anionic lipids, including PS, PA, and PI, result in a net negative surface charge, which is essential for interactions with the cytoskeleton and numerous intracellular signaling proteins (Takenawa and Itoh, 2001; Yeung et al., 2008). Although representing only approximately 1% of the total phospholipids in the inner leaflet, phosphatidylinositol phosphates, particularly PIP_2_, play disproportionately important roles in regulating ion channels and diverse signal transduction pathways (Huang, 2007; Rodríguez-Menchaca et al., 2012; Harraz et al., 2020).

**FIGURE 1:**
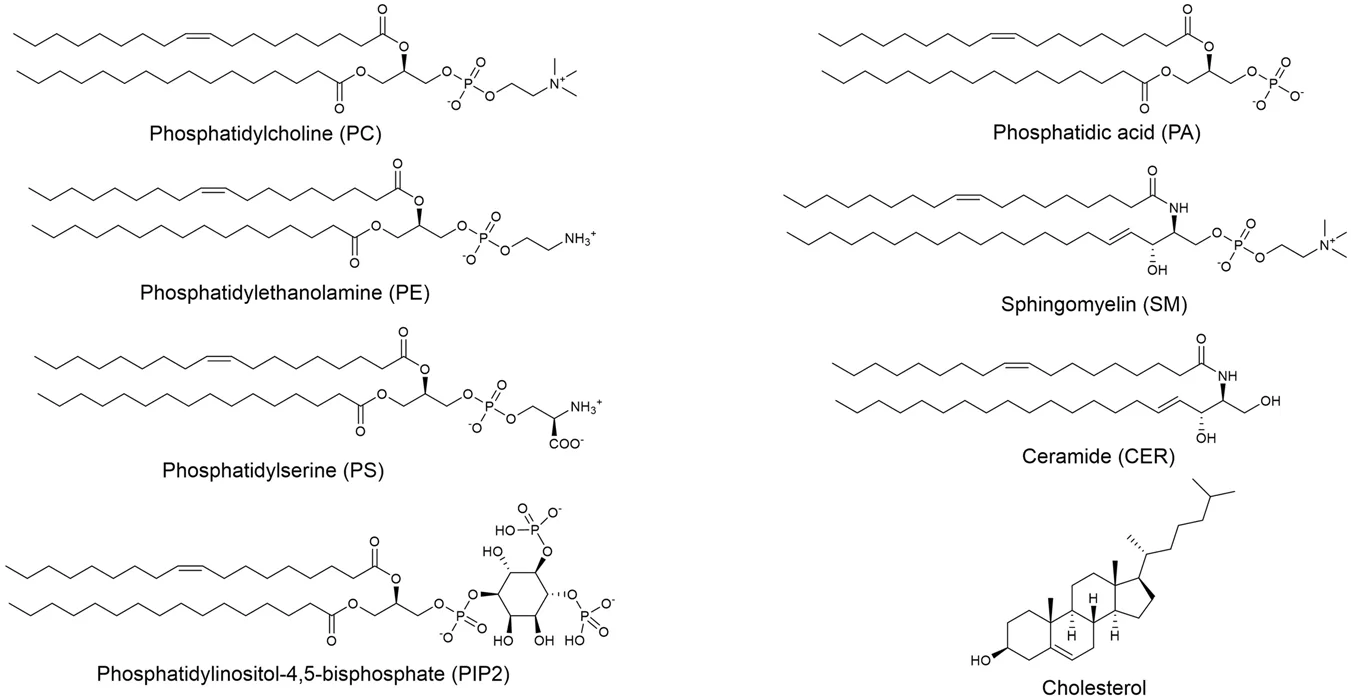
Representative structures of common plasma membrane lipids.

Cholesterol is the most abundant lipid species in the mammalian plasma membrane, accounting for approximately 30–50 mol% (Ingólfsson et al., 2014). Unlike some phospholipids, cholesterol distributes across both leaflets, where it acts as a dynamic glue that modulates key biophysical properties of the membrane. Cholesterol influences lipid packing, membrane thickness, permeability, and phase behavior, and is a major determinant of the formation of liquid-ordered (L_o_) membrane domains (Simons and Ehehalt, 2002; Szlasa et al., 2020). The structures of cholesterol and the major membrane lipids are illustrated in Figure 1.

In the plasma membrane, lipid constituents are not uniformly distributed but instead self-organize into lipid rafts, which are specialized microdomains with distinct biochemical compositions and biophysical properties. Lipid rafts are typically small (10–200 nm), dynamic, and heterogeneous domains enriched in cholesterol and sphingolipids, and they exist in the liquid-ordered (L_o_) state. These domains coexist with the surrounding liquid-disordered (L_d_) phase, which is primarily composed of unsaturated phospholipids. Although individual rafts assemble and disperse rapidly, they can merge into larger platforms to support specific cellular processes (Owen et al., 2012; Tsuchiya and Mizogami, 2020). Recent advances in fluorescence and super-resolution microscopy have revealed the essential roles of lipid rafts in signal transduction, intracellular trafficking, endocytosis, and in the formation of caveolae, which are small (50–100 nm) invaginations of the plasma membrane (Owen et al., 2012; Owen and Gaus, 2013; Anselmo et al., 2025). Moreover, lipid rafts act as key sites for lipid–protein interactions and have been implicated in a range of pathological conditions, including neurological disorders, cardiovascular diseases, cancer, and infectious diseases (Lucero and Robbins, 2004).

### 2.2 Lipid composition of cancer cell membrane

Unlike physiological states, neoplastic cells undergo extensive remodeling of the plasma membrane, resulting in fundamentally altered lipid compositions (Szlasa et al., 2020). However, it is important to note that this remodeling is not uniform across all malignancies but significantly heterogeneous regarding lipid composition, microdomain organization, and the magnitude of specific alterations across different cancer types and stages (Bernardes and Fialho, 2018; Pakiet et al., 2019). Despite this variability, several features of cancer-associated membrane remodelling have been consistently identified.

A major characteristic is the disruption of transbilayer lipid asymmetry. Under normal conditions, the anionic phospholipid, phosphatidylserine (PS), is strictly distributed within the cytosolic leaflet (Calianese and Birge, 2020). However, in various neoplastic cells, PS becomes externalized to the exoplasmic leaflet (Birge et al., 2016). Unlike apoptosis-induced PS exposure, this externalization in cancer cells is actually a pro-survival mechanism (Lankry et al., 2013). The presence of PS on the outer surface also alters the surface charge density, i.e., shifting it towards a more negative potential, thereby modulating interactions with the extracellular matrix and the immune system, influencing local ion concentrations, and affecting lipid-protein interactions that regulate ion channels and receptors (Szlasa et al., 2020). This abnormal PS exposure is widespread among tumor cells and contributes significantly to immune evasion, metastatic potential, and enhanced cell proliferation (Riedl et al., 2011). In addition, alterations in the abundance and distribution of other anionic lipids, particularly phosphatidylinositol-(4,5)-bisphosphate (PIP_2_), have been documented, implicating crucial intracellular signaling pathways (Mandal, 2020).

Another notable feature of neoplastic membranes is the change of cholesterol and sphingolipids, particularly within lipid rafts and other microdomains. For example, reduction of membrane cholesterol could help cancer cells facilitate metastasis by increasing membrane fluidity and plasticity required for vascular invasion (Zalba and Ten Hagen, 2017). On the other hand, enrichment of cholesterol levels promotes raft reorganization and shifts the balance toward the liquid-ordered (L_o_) phase, thereby increasing membrane thickness, rigidity, and lipid packing density (Kuzu et al., 2016). In cancer cells, lipid rafts and caveolae often exhibit upregulation in both density and functional activity (Codini et al., 2021). Notably, cancer stem cells exhibit significantly higher lipid raft levels compared to non-malignant cells (Zhang et al., 2022). These microdomains function as organizational platforms for the aggregation of receptors, kinases, and ion channels. Abnormal raft dynamics could result in the spatial reorganization of membrane proteins, modifying their intermolecular interactions and regulatory functions to favor tumorigenesis (Codini et al., 2021). Moreover, evidence suggests that the structural stratification and cholesterol interactions within tumor-associated rafts differ intrinsically from those in healthy tissue (Mollinedo and Gajate, 2020). Additionally, cancer cells frequently exhibit an increased degree of fatty acid saturation (reduced unsaturation). This alteration enhances the stability of lipid raft domains, promoting more robust protein-membrane association and conferring protection against lipid peroxidation induced by reactive oxygen species (ROS). However, it is noteworthy that increased saturation is not a universal feature across all cancer types (Szlasa et al., 2020).

**FIGURE 2:**
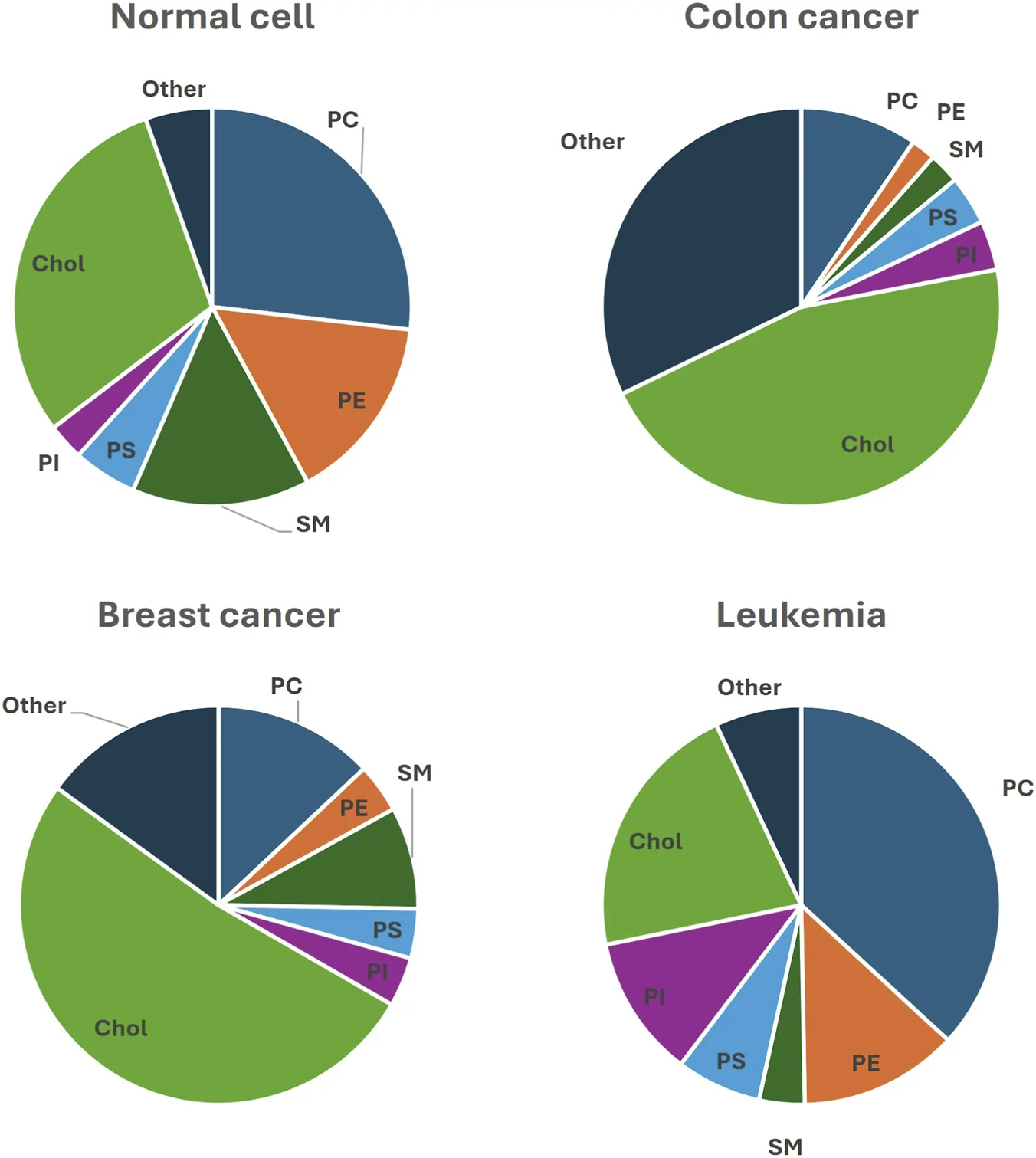
Comparative lipid composition of normal and cancer cell membranes.

A comparative analysis of membrane lipid composition between normal cells and selected malignancies, including colorectal cancer, breast cancer, and leukemia, is presented in Figure 2 (Zech et al., 2009; Ingólfsson et al., 2014; Szlasa et al., 2020). In normal cells, cholesterol is the predominant component (29.9%), followed by phosphatidylcholine (PC, 26.0%), phosphatidylethanolamine (PE, 15.2%), and sphingomyelin (SM, 14.4%), with minor contributions from phosphatidylserine (PS), phosphatidylinositol (PI), and diacylglycerol (DAG). In contrast, cancer cells exhibit distinct lipidomic signatures that reflect their metabolic and functional adaptations. Colorectal cancer cells demonstrate a marked increase in cholesterol (45.0%) and ether lipids, specifically PE ether (7.3%) and PC ether (16.8%), accompanied by a reduction in PC (9.5%) and SM (2.5%). Similarly, breast cancer cells show the highest cholesterol content among the malignancies examined (49.5%) and elevated PC ether (10.5%), while maintaining moderate PC (13.0%) and SM (8.3%) levels. On the other hand, leukemic cells present a unique lipid distribution characterized by significantly increased PC levels (40.0%) and PI (12.5%), while cholesterol levels are reduced to 23%. These alterations are not only relevant for membrane structure and signaling but may also influence the behavior of membrane proteins embedded within this environment, including ion channels. In particular, changes in lipid composition, membrane order, and microdomain organization may modulate channel activity, localization, and interactions with regulatory partners. Despite this, the impact of cancer-associated lipid remodeling on potassium channel function, and especially on Kv10.1, remains largely unexplored.

Although direct experimental evidence linking cancer-associated membrane lipid remodeling to altered Kv10.1 function is currently lacking, several lipid species that are consistently remodeled in tumors, including PIP2, cholesterol, and polyunsaturated fatty acids, are established regulators of Kv10.1. This convergence suggests a plausible mechanistic framework in which changes in membrane composition may modify Kv10.1 gating, localization, and pharmacological behavior. Evaluating this hypothesis first requires understanding the structural organization of Kv10.1 and how it differs from Kv11.1, as these structural features provide the basis for their distinct lipid sensitivities.

## 3 Overview of Kv10.1 channel

### 3.1 Structure of Kv10.1 channel

**FIGURE 3:**
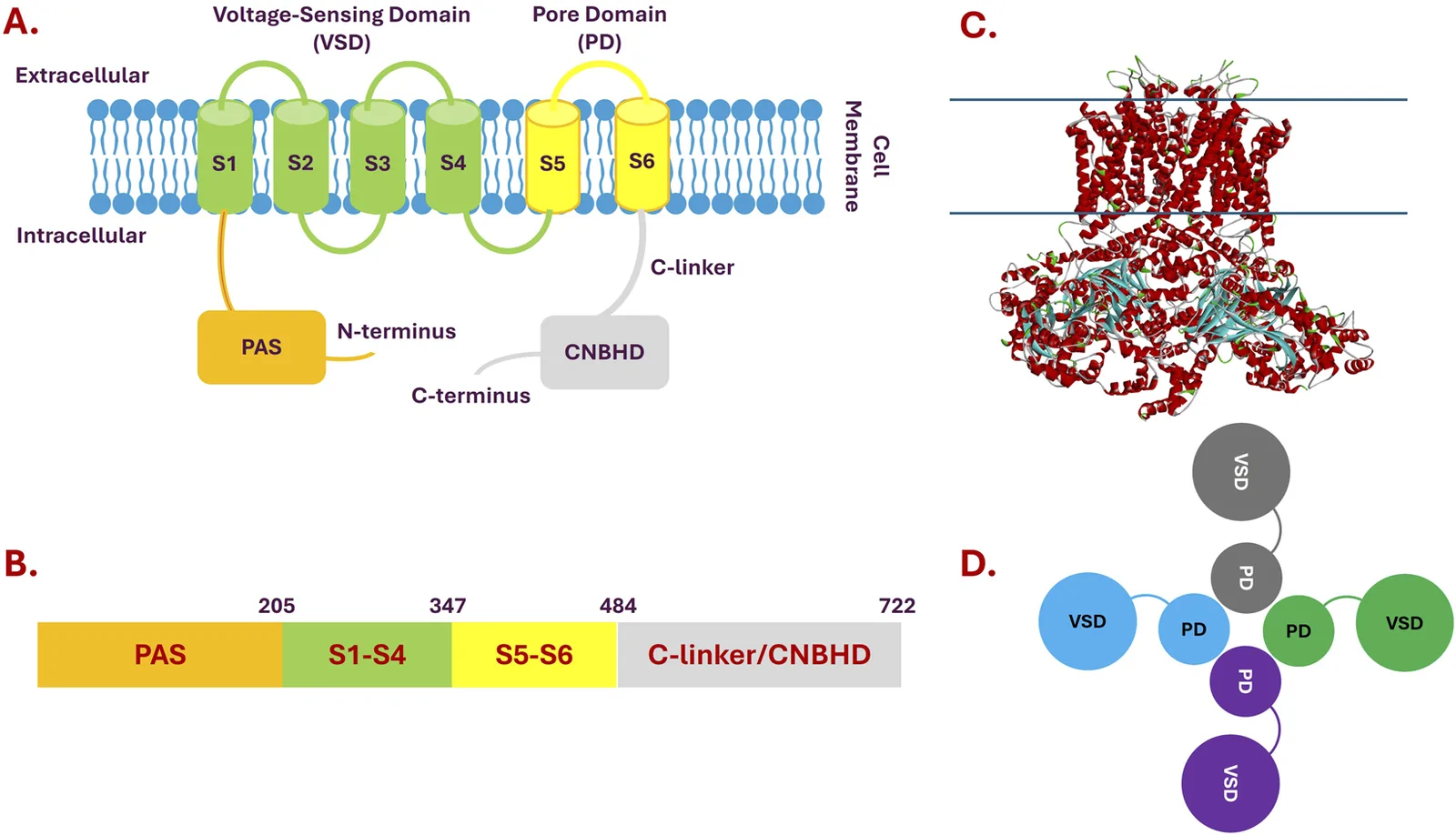
Overview of the structure of Kv10.1 channel. **(A)** Topological map of Kv10.1. **(B)** Linear amino acid segmentation. **(C)** Cryo-EM structure of Kv10.1 in complex with calmodulin (PDB ID: 5K7L). **(D)** Schematic representation of the non-swapping domain architecture.

Kv10.1 (also known as Eag1) is a member of the KCNH (ether-à-go-go) family and is characterized by a complex topology comprising multiple transmembrane segments and extensive intracellular domains that critically regulate channel gating (Cázares-Ordoñez and Pardo, 2017). Like other voltage-gated potassium channels, Kv10.1 assembles as a homotetramer, with each subunit containing six transmembrane segments (S1–S6). Segments S1 through S4 form the voltage-sensing domain (VSD), in which S4 contains positively charged amino acids responsible for detecting changes in membrane potential. Depolarization-induced movement of S4 triggers conformational changes that transition the channel between its closed and open states. Segments S5 and S6 constitute the pore domain (PD), which includes a central aqueous cavity and the ion selectivity filter (Whicher and MacKinnon, 2016). Notably, previous studies revealed that the pore domains of Kv10.1 and Kv11.1 (hERG) share high sequence homology and strong structural conservation, making the pore region a particularly challenging target for developing selective Kv10.1 inhibitors (Luis et al., 2022). Another feature of Kv10.1 shared with other members of the KCNH family, such as Kv11.1, is its non-domain-swapped architecture. Unlike Shaker-type Kv channels, where the VSD of one subunit interacts with the PD of a neighboring subunit, the VSD in Kv10.1 interacts directly with the PD of the same subunit (Figure 3). This arrangement, together with an unusually short S4–S5 linker, contributes to a unique gating mechanism that differentiates Kv10.1 from other voltage-gated channels (Whicher and MacKinnon, 2016; Tomczak et al., 2017; Abdelaziz et al., 2024).

Beyond the transmembrane region, Kv10.1 (and Kv11.1) also has large intracellular domains that account for a significant proportion of the channel’s mass. These intracellular parts include an N-terminal Per-Arnt-Sim (PAS) domain and a C-terminal Cyclic Nucleotide-Binding Homology Domain (CNBHD), which is connected to the S6 segment via a C-linker (Luis et al., 2022). Despite structural similarities to the CNBHD of other potassium channels, the CNBHD of Kv10.1 lacks affinity for cAMP or cGMP. Instead, the PAS domain interacts directly with the CNBHD to form a regulatory complex beneath the VSD, thereby modulating the channel gating. Kv10.1 also undergoes glycosylation at residues Asn-388 and Asn-406. This post-translational modification is critical for the trafficking of the channel to the plasma membrane and the maintenance of optimal current density. Removal of these glycan moieties has been shown to significantly reduce channel activity (Whicher and MacKinnon, 2016).

### 3.2 Overview of Kv10.1 inhibitors

Up until now, there has been no Kv10.1 inhibitor that has met the criteria required to advance into clinical trials. Nevertheless, numerous compounds are still under investigation. In this review, we focus exclusively on small molecules. Readers interested in peptide or antibody-based inhibitors are referred to detailed reviews elsewhere (Toplak et al., 2022). The chemical structures of the small molecules discussed in this section are summarized in Table 1.

**TABLE 1:** Summary of some compounds targeting Kv10.1.

Binding site	Compound	Structure	IC_50_ on Kv10.1	IC_50_ on Kv11.1	Interacted residues	Ref.
Pore domain	Quinidine	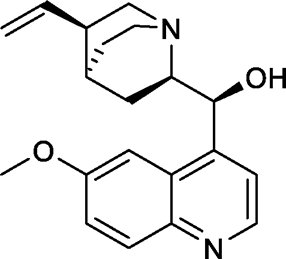	2.08 ± 0.36 μM	2.50 ± 1.27 μM	Phe468	Gessner et al. (2004), Gillie et al. (2013)
	Clofilium	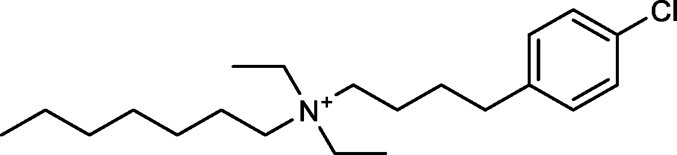	255 ± 35 nM	2.59 ± 0.71 nM	Ser436, Val437, Ala453	Gessner and Heinemann (2003)
	Dofetilide	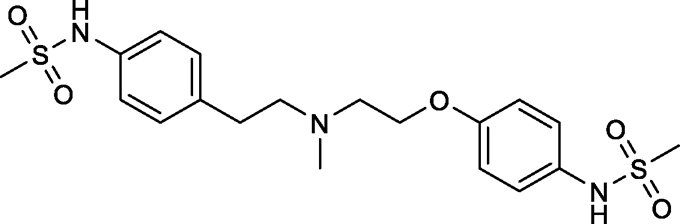	29.6 ± 1.1 μM	0.32 ± 0.04 μM	Phe468	Ficker et al. (1998), Gómez-Varela et al. (2006)
	Astemizole	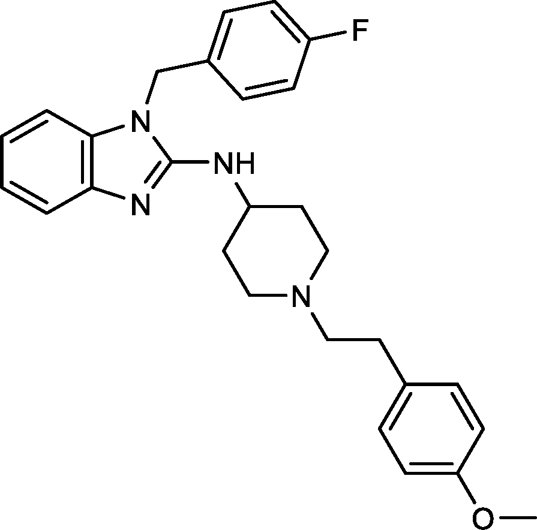	2.8 ± 0.1 μM	48.4 ± 3.8 nM	Phe468	Suessbrich et al. (1996), Gómez-Varela et al. (2006)
Voltage sensor domain	Amiodarone	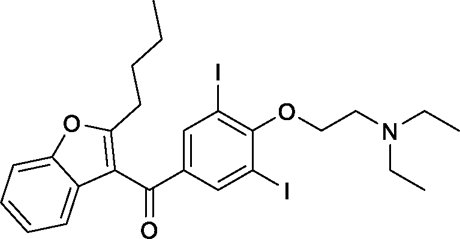	K_d_ = 203 nM	0.22 µM	-	Waldhauser et al. (2008), Barriga-Montoya et al. (2018)
	Ginsenoside 20(S)-Rg3	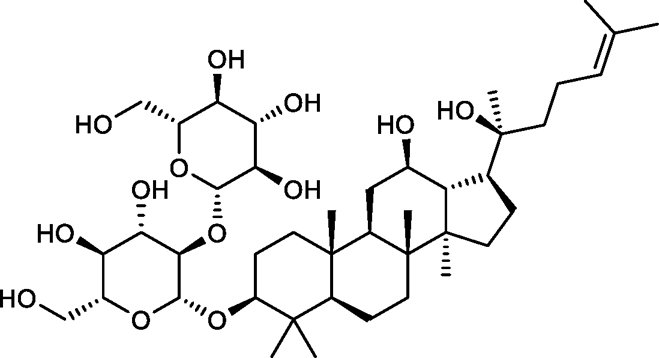	1.31 ± 0.16 μM	255 ± 34 nM	-	Wu et al. (2016)
	Corydaline	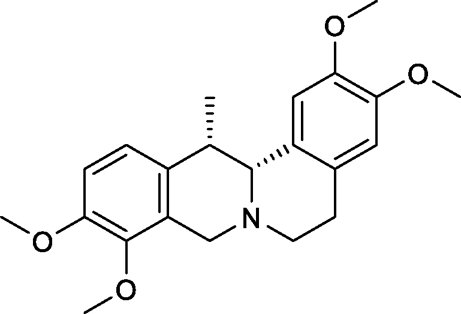	11.3 ± 0.6 μM	111.4 ± 8.5 μM	Lys217, Phe273, Pro276, Trp295, Arg366	Ma et al. (2024b)
PAS domain	Chlorpromazine	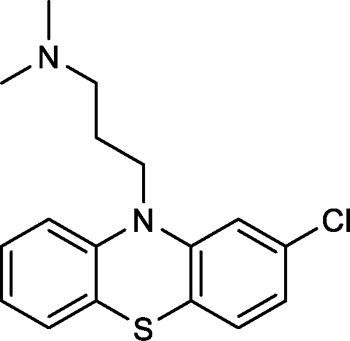	3.7 ± 0.7 µM	21.6 ± 6.9 µM	-	Thomas et al. (2003), Wang et al. (2020)
	Imipramine	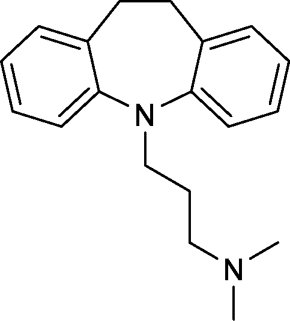	40.2 ± 0.3 μM	3.4 ± 0.4 μM	Asp39, Arg84	Teschemacher et al. (1999), Wang et al. (2023)
	Undecylenic acid	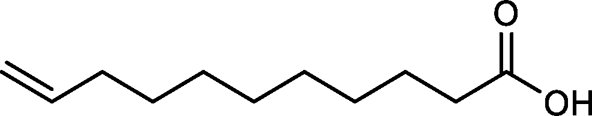	1.1 ± 0.02 μM	No binding to the PAS and C-linker/CNBHD of hERG at 100 μM	-	Wang Z.-J. et al. (2019)
C-linker/CNBHD	Tetrandrine	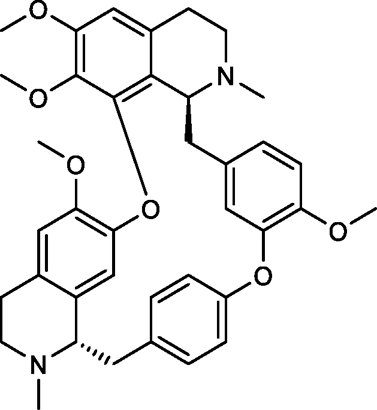	69.97 ± 5.24 μM	>100 μM	Ile550, Thr552, Gln557	Wang X. et al. (2019)
	Procyanidin B1	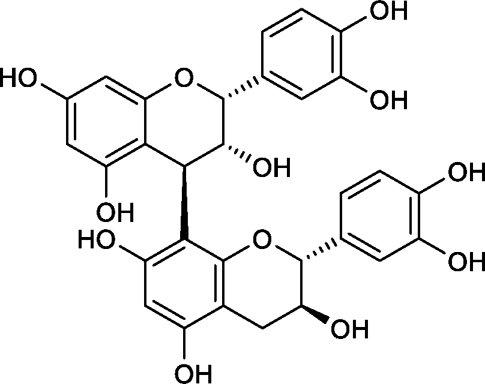	10.38 ± 0.87 μM	No significant activity against hERG at 100 μM	Ile550, Thr552 Gln557	Na et al. (2020)
	Liensinine	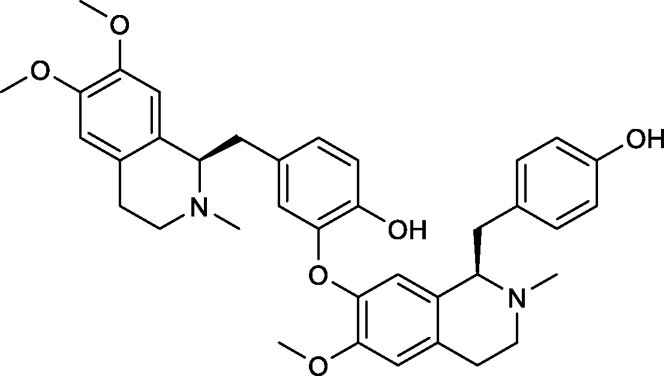	0.24 ± 0.07 μM	67.0 ± 11.1 *μ*M	Tyr539, Thr543, Asp551, Glu553, His601	Ma et al. (2024a)

As can be seen from Table 1, while pore-targeting compounds show certain diversity in their structure, they still share a common pharmacophore: an alkyl amine or piperidine derived scaffold. This feature seems to be critical for pore blockade activity. Additionally, several molecules contain sulfonamide moieties. Previous studies have shown that this group plays a critical role in hERG inhibitory activity by forming essential hydrogen bonds with residues within the channel’s pore domain (Maly et al., 2022). Given the high sequence similarity between the pore domains of Kv11.1 and Kv10.1 (Toplak et al., 2022), it is reasonable to assume that sulfonamides contribute similarly to the inhibition of Kv10.1. However, this conservation also presents a significant pharmacological challenge. Due to the similarity in the PD, most pore-blocking compounds exhibit substantial cross-reactivity, often binding Kv11.1 with even higher affinity than Kv10.1. As a result, strategies relying solely on pore blockade must be performed with extreme caution due to the associated risk of cardiotoxicity.

In contrast, targeting regions outside the pore, such as the Voltage-Sensor Domain (VSD) or the Per-Arnt-Sim (PAS) domain, has yielded more promising results regarding selectivity. For example, corydaline, a VSD inhibitor, shows an IC_50_ of 11.3 μM for Kv10.1 compared to 111.4 μM for hERG, achieving an approximate 10-fold selectivity (Ma et al., 2024b). Similarly, chlorpromazine, which targets the PAS domain, demonstrates mild selectivity with an IC_50_ of 3.7 ± 0.7 µM for Kv10.1 versus 21.6 ± 6.9 µM for Kv11.1 (Thomas et al., 2003; Wang et al., 2020). Using a surface plasmon resonance-based screening method to identify PAS domain ligands, Brelidze et al. identified undecylenic acid as a potent Kv10.1 inhibitor (IC_50_ = 1.1 μM). Notably, this compound showed no detectable binding to Kv11.1 under the study conditions (Wang Z.-J. et al., 2019). Regarding structure, VSD ligands tend to be more diverse and bulkier compared to pore blockers, whereas PAS-domain inhibitors, with the exception of undecylenic acid, commonly share the tricyclic scaffold, which is found in many antidepressant drugs (Table 1).

The results from Table 1 also show that the C-linker/Cyclic Nucleotide-Binding Homology Domain (CNBHD) is a particularly attractive site for achieving selective Kv10.1 inhibition. Current inhibitors targeting this region, including tetrandrine, procyanidin B1, and liensinine, are all natural products with bulky polycyclic structures. These compounds show the strongest selectivity profiles reported thus far. Liensinine, for instance, inhibits Kv10.1 with an IC_50_ of 0.24 μM while showing significantly weaker activity on Kv11.1 (IC_50_ ≈ 67 μM), corresponding to a selectivity ratio of approximately 280-fold (Ma et al., 2024a). Furthermore, procyanidin B1 and tetrandrine similarly demonstrate potent Kv10.1 inhibition with negligible effects on hERG (Wang X. et al., 2019; Na et al., 2020). Interestingly, a recent study demonstrated that certain structurally simpler flavonoids, such as luteolin and fisetin, could also bind well to the CNBHD but act as Kv10.1 activators rather than inhibitors (Carlson et al., 2013). It is noteworthy that the CNBHDs of Kv10.1 and Kv11.1 share a very high degree of similarity (Whicher and MacKinnon, 2016). Therefore, we assume that the observed selectivity is likely influenced by the differences in the interactions between CNBHDs with other channel components, such as the N-cap domain (Haitin et al., 2013). While the precise molecular mechanisms remain incompletely understood, current evidence suggests that the CNBHD may be a promising allosteric site for developing therapeutics capable of discriminating effectively between Kv10.1 and hERG.

Having outlined the structural organization of Kv10.1 and the pharmacological challenges associated with achieving selectivity over Kv11.1, we next examine the lipid-dependent regulatory mechanisms that provide the basis for the conceptual framework proposed in this review.

## 4 Lipid modulation of Kv10.1 channels

Previous studies have shown that Kv10.1 activity is regulated by various factors, including divalent cations (such as Ca^2+^ and Mg^2+^) and a complex network of interacting proteins (e.g., K^+^ channel regulator 1 (KCR1), CaM-dependent kinase, the 14-3-3 proteins, epsin, rabaptin-5, and cortactin) (Han et al., 2017). In addition to these protein interactions, specific membrane lipids, such as phosphatidylinositol 4,5-bisphosphate (PIP_2_), cholesterol, and polyunsaturated fatty acids (PUFAs), have been identified as critical modulators of Kv10.1 (Han et al., 2017). The comparative overview of the lipid regulation of Kv10.1 and Kv11.1 is summarized in Table 2.

**TABLE 2:** Direct comparison of structure, pharmacology and lipid regulation of Kv10.1 and Kv11.1.

Feature	Kv10.1	Kv11.1
Structure	Homotetramer; each subunit contains an N-terminal PAS domain, VSD, pore domain, C-linker and CNBHD. Non-domain-swapped organization	Same conserved KCNH-domain organization and non-domain-swapped topology
Characteristic gating	Relatively little rapid inactivation; activation is strongly modulated by intracellular domains, CaM and membrane lipids	Rapid selectivity filter-mediated (C-type-like) inactivation and exceptionally slow deactivation
Drug sensitivity	- Many pore blockers of Kv10.1 also inhibit Kv11.1 well because of pore conservation - VSD, PAS and C-linker/CNBHD ligands can provide better selectivity	
PIP_2_ regulation	- A complex, likely biphasic, dependence on PIP2 concentration - A short segment near the CaM-binding site (N-terminus) is required for PIP_2_ binding and regulation	- PIP_2_ increases current, facilitates activation and modifies inactivation. PIP_2_ depletion suppresses channel function - A C-terminal polycationic region is implicated in PIP_2_ binding and functional regulation
Cholesterol and lipid raft regulation	- Cholesterol depletion increases current density without altering activation kinetics - The EAG1 structure 5K7L contains cholesterol hemisuccinate/sterol-like density at the VSD-pore domain interface - Approximately 60% of Kv10.1 was resided in cholesterol- and sphingolipid-rich detergent-resistant membranes and approximately 40% in non-DRM fractions. The DRM-associated population was proposed to be relatively electrically silent	- Cholesterol changes hERG function through indirect mechanisms - No equivalent experimentally validated sterol site has been identified in Kv11.1 structure - Kv11.1 has also been detected in cholesterol- and sphingolipid-enriched membrane fractions and is associated functionally with caveolin-containing domains
PUFAs regulation	Arachidonic acid activates Kv10.1, lowers its activation threshold, accelerates activation and increases open probability Sided application of arachidonoyl-CoA suggests an interaction accessible from the extracellular leaflet, but the molecular site is unknown Undecylenic acid is mechanistically different and inhibits Kv10.1 by binding the intracellular PAS region	- AA and DHA inhibit Kv11.1 via direct lipophilic interaction with the channel - The precise PUFA-binding site is unknown but likely to be the VSD and pore domain

### 4.1 Phosphatidylinositol 4,5-bisphosphate (PIP2)

Phosphatidylinositol 4,5-bisphosphate (PI(4,5)P_2_ or PIP_2_) is an anionic phosphoinositide that accounts for less than 1% of total cellular phospholipids and is predominantly localized to the inner leaflet of the eukaryotic plasma membrane (Czech, 2000). With a bis-phosphorylated inositol headgroup, PIP_2_ exhibits a high negative charge density, enabling strong electrostatic interactions with clusters of basic amino acids on membrane proteins. In addition, PIP_2_ is a precursor for multiple important second messengers such as IP_3_, DAG, and PIP_3_ through kinase- and phosphatase-mediated pathways (Lentini et al., 2025; Luong et al., 2025). It is widely regarded as a “master regulator” of membrane transport and signaling, modulating hundreds of membrane-associated proteins (Gada and Logothetis, 2022). PIP_2_ regulates transmembrane proteins through two major mechanisms: (1) direct binding to and regulation of protein targets via specific binding sites; and (2) indirect effects arising from its metabolic conversion into signaling intermediates that alter intracellular Ca^2+^ levels or activate PKC and PI3K pathways (Yaradanakul et al., 2007; Tariq and Luikart, 2021). Numerous studies have established PIP_2_ as an essential cofactor for a broad range of ion channels and transporters, such as Na^+^/Ca^2+^ exchangers, K_ATP_ (ATP-sensitive potassium) channels, TRP (Transient Receptor Potential) channels, voltage-gated Ca^2+^ and Na^+^ channels, and Cl^−^ channels (Hilgemann et al., 2001; Yaradanakul et al., 2007; Tembo et al., 2019; Pipatpolkai et al., 2022; Huang and Chen, 2025).

Among potassium channels, PIP_2_ has been shown to regulate a wide range of members, such as Kir6.2, Kir2.1, Kv11.1 (hERG), and Kv7.1 (KCNQ1) (Bian et al., 2001; Ribalet et al., 2005; Xie et al., 2008; Zaydman et al., 2012). Regarding Kv11.1, PIP_2_ acts as an obligatory cofactor required for proper channel function (Bian et al., 2001; Rodriguez et al., 2010). Increase of intracellular PIP_2_ concentrations enhances hERG activity, increasing current density, accelerating activation kinetics, and slowing inactivation (Bian et al., 2001; Wang et al., 2004). The intracellular delivery of 10 μM PIP_2_ into hERG-expressing CHO/HEK293 cells was demonstrated to induce a 20%–80% increase in current amplitude and a hyperpolarizing shift in the voltage-dependence of activation by approximately 10–19 mV. On the other hand, sequestration of endogenous PIP_2_ using specific monoclonal antibody (PIP_2_-Ab, 60 nM) significantly reduced current density and induced a depolarizing shift in activation (V_1/2_ from 18.1 ± 1.6 mV to 11 ± 2.2 mV), confirming that PIP_2_ depletion suppresses Kv11.1 activity (Bian et al., 2001; Bian et al., 2004). Previous studies also revealed that PIP_2_ modulates Kv11.1 by binding directly to a polycationic region (residues 883–894) located at the C-terminus of the channel (Bian et al., 2004; Bian and McDonald, 2007).

Kv10.1 is also strongly regulated by PIP_2_, but its response is fundamentally distinct from the stabilizing action observed in Kv11.1. Available evidence suggests a complex, likely biphasic, dependence on PIP_2_ concentration. Rodríguez-Menchaca *et al.* showed that exogenous application of 5 μM PIP_2_ inhibited hEAG1 activity by 64.6% ± 3.4%. Similarly, PIP_2_ depletion induced by the activation of the human muscarinic type-1 receptor (hM1R) or the voltage-sensing phosphatase from *Danio rerio* (Dr-VSP) also inhibit Kv10.1 channel activity (Delgado-Ramírez et al., 2018). However, conflicting results were reported by Hou et al., where PIP_2_ reduction via rapamycin or serotonin treatment led to an increase in hEAG1 currents (Han et al., 2016). Because exact PIP_2_ concentrations were not determined in these studies, it is still reasonable to assume that Kv10.1 operates optimally within a narrow PIP_2_ concentration window, with deviations on either side leading to inhibition. In this study, Hou et al. also determined the IC_50_ of native PIP_2_ to be 0.35 ± 0.01 μM, compared to 18.6 ± 0.6 μM for the short-chain analog diC8-PIP_2_, suggesting that the acyl chain length is a key determinant of inhibitory potency. Comparative analysis with other phosphoinositides revealed that PIP_2_ and PI(3,5)P_2_ exhibit the strongest inhibition, whereas unphosphorylated PI had no activity, indicating the necessity of the anionic headgroup for Kv10.1 modulation (Han et al., 2016).

Interestingly, the structural basis for PIP_2_ interaction differs significantly between the two channels. Whereas PIP_2_ modulates Kv11.1 by binding to its C-terminal polycationic region, deletion of the corresponding positively charged segment in Kv10.1 does not alter PIP_2_-mediated inhibition. Through mutagenesis of the S4 segment and N-/C-terminal calmodulin (CaM) binding sites, it was determined that PIP_2_ binds directly to a short segment near the CaM-binding site of the Kv10.1 N-terminus (Han et al., 2016). This interaction shifts the voltage dependence of activation, providing a mechanistic explanation for its inhibitory action. These distinct binding sites and functional response to PIP_2_ could be exploited for designing inhibitors with high selectivity for Kv10.1 over Kv11.1.

### 4.2 Cholesterol and lipid raft

Cholesterol is preferentially concentrated within sphingolipid-rich microdomains of the plasma membrane, commonly known as lipid rafts. Owing to its rigid, planar sterane backbone and hydrophobic tail, cholesterol inserts between neighboring phospholipids, where it restricts the movement of unsaturated acyl chains while preventing the crystallization of saturated ones (Simons and Ehehalt, 2002). Through these interactions, cholesterol plays a central role in modulating membrane order, thickness, and curvature. Cholesterol-rich microdomains act as organizational scaffolds for numerous transmembrane proteins, particularly receptors and ion channels, facilitate their assembly into signaling complexes, and regulate both protein–protein interactions and signal transduction dynamics (Tsuchiya and Mizogami, 2020).

Mechanisms of cholesterol-mediated regulation can be broadly categorized as direct or indirect (Zakany et al., 2020). Direct regulation involves specific binding of cholesterol to defined protein motifs, such as CRAC or CARC sequences (Fantini and Barrantes, 2013). This mode of action displays sterol specificity: substituting cholesterol with its analogues that have similar effects on the membrane’s properties (e.g., epi-cholesterol) typically fails to reproduce its physiological effects, indicating that cholesterol functions as a “lipid ligand” within a hydrophobic binding pocket (Levitan et al., 2014; Valdivia et al., 2023). In contrast, indirect regulation arises from cholesterol-driven changes in membrane biophysics, including alterations in bilayer thickness, lateral pressure, curvature, and phase behavior, or from the spatial segregation of proteins into or out of raft domains. High cholesterol levels favor the formation of ordered, raft-like membranes that cluster palmitoylated or raft-preferring proteins, whereas PIP_2_ often redistributes other proteins away from cholesterol-rich regions, creating a dynamic nanoscale antagonism between these two lipids (Levitan et al., 2010; Beverley and Levitan, 2024). Regarding the role of lipid rafts in cancer cells, Paul S. Mischel et al. found that the stabilization of lipid rafts in tumors could promote the enrichment of EGFR within these membrane microdomains, therefore, sustains EGFR phosphorylation and activates downstream signaling pathways, including PI3K/AKT and mTOR, which support cancer cell growth and survival. In contrast, disrupting lipid raft integrity and decreasing membrane order will cause EGFR to dissociate from these microdomains. As a result, cancer cell proliferation declines while cell death increases (Bi et al., 2019).

In the case of Kv11.1 (hERG), available evidence suggests a predominantly indirect regulatory mechanism driven by cholesterol-dependent activation of phospholipase C (PLC), which in turn reduces PIP_2_ levels. High cholesterol levels increase the surface expression of PLCβ1 and PLCβ3, leading to the suppression of PIP_2_-sensitive channels, including hERG. On the other hand, hERG activity can be restored by intracellular application of PIP_2_ or pharmacological inhibition of PLC. Furthermore, cholesterol depletion with methyl-β-cyclodextrin (MβCD) or cholesterol loading in HEK293 cells likewise alters hERG function (Chun et al., 2010). A clinical example of this relationship is observed with probucol, a lipid-lowering agent associated with long QT syndrome. Gou et al. demonstrated that probucol does not directly block the hERG pore; instead, it reduces hERG expression via a unique trafficking mechanism. Specifically, probucol-induced cholesterol reduction accelerates the degradation of caveolin-1 (Cav-1), a cholesterol-binding scaffolding protein. Since hERG interacts with caveolin-1, this turnover would destabilize the hERG-Cav-1 complex and lead to a downregulation of the channel. Cav-1 overexpression was observed to enhance the pharmacological effect of probucol on Kv11.1, while Cav-1 silencing revoked it. In addition, low-density lipoprotein, a highly effective cholesterol transporter, was able to prevent the probucol activity on hERG in hERG-HEK cells and in cultured neonatal rat ventricular myocytes (Guo and Zhang, 2011). In another study, Annarosa Arcangeli et al. demonstrated that in pancreatic adenocarcinoma cells, hERG associate with β1-integrin within the cholesterol-rich membrane microdomains (lipid raft) to form hERG/β1-integrin signaling complexes, converting hERG from a simple ion-conducting channel into a regulator of oncogenic signaling. This complex activates the PI3K/Akt pathway, thereby promoting cancer cell proliferation and migration. Disruption of lipid rafts through cholesterol depletion with methyl-β-cyclodextrin or inhibition of cholesterol synthesis using statins destabilizes the hERG/β1-integrin complex, resulting in reduced activation of PI3K/Akt and Rac1 signaling. As a result, both cancer cell proliferation and migration are suppressed. Notably, combining statins with antibodies targeting the hERG1/β1-integrin complex produces a synergistic inhibitory effect on tumor growth, which demonstrate that lipid rafts are essential for the oncogenic signaling functions of hERG1 and highlight their potential as therapeutic targets in pancreatic cancer (Duranti et al., 2025).

**FIGURE 4:**
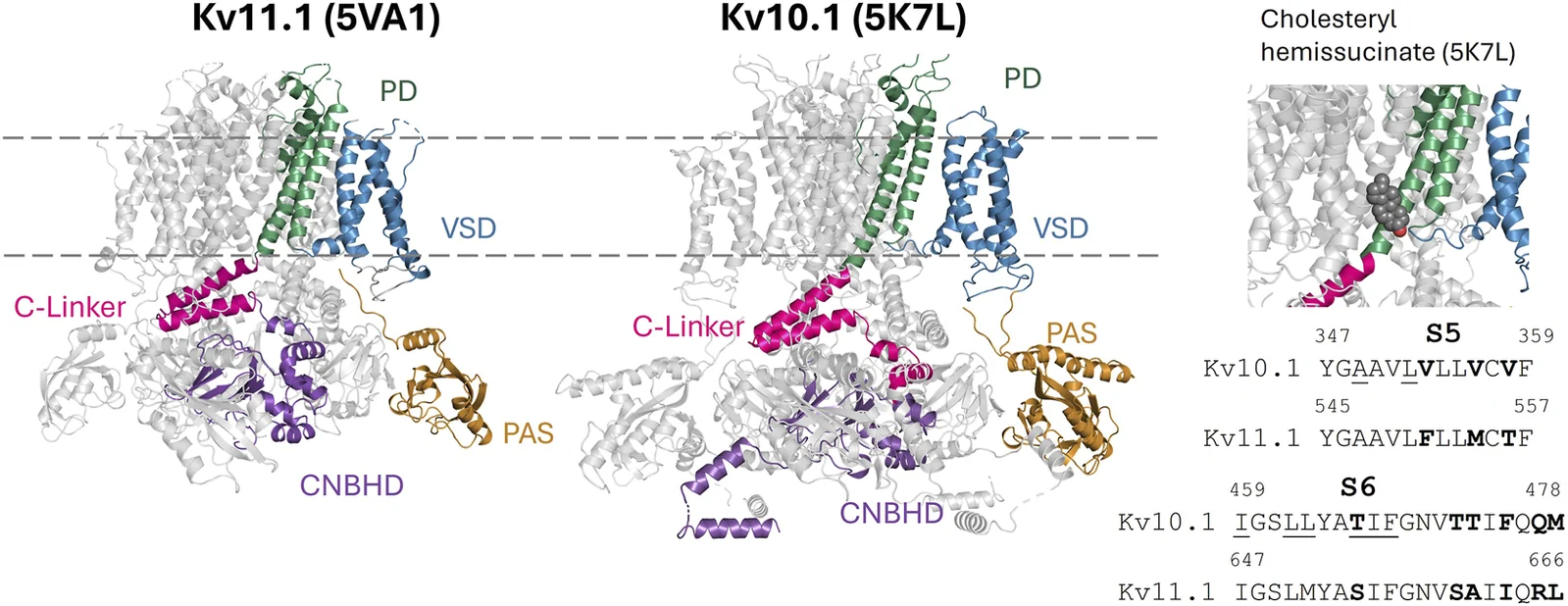
Structural comparison of Kv10.1 and Kv11.1 domain organization. Representative subunits of Kv10.1 (right) and Kv11.1 (left) are shown with the voltage-sensing domain (VSD, blue), pore domain (PD, green), PAS domain (orange), C-linker (magenta), and cyclic nucleotide-binding homology domain (CNBHD, purple) highlighted. The remaining subunits are shown in light grey for context. The structures were structurally aligned and are displayed in the same orientation and at identical scale; the aligned models were translated horizontally for clarity. Approximate membrane boundaries are indicated by dashed lines. The inset highlights a putative cholesteryl hemisuccinate (CHS) binding pocket observed in the Kv10.1/EAG1 structure (PDB 5K7L). Underlined residues in the local S5 and S6 alignments correspond to Kv10.1 residues contacting CHS.

Regarding Kv10.1, Ortega et al. reported that these channels partition into two membrane domains in an approximate 3:2 ratio: ∼60% reside within cholesterol- and sphingolipid-rich detergent-resistant membranes (DRMs), while the remaining channels localize to non-raft regions. When reducing the total membrane cholesterol by 81% (from 0.462 to 0.09 mg/mg protein) using 30 mM MβCD for 1 h, the current density of Kv10.1 significantly increased without altering activation kinetics. These findings suggest that the DRM-resident population of Kv10.1 represents a “silent” or reserve pool that becomes electrically active upon release from the raft environment (Jimenez-Garduno et al., 2014). While the precise molecular mechanism remains to be fully elucidated, structural studies provide an interesting point of comparison between Kv10.1 and Kv11.1. In the Kv10.1 cryo-EM structure (PDB ID: 5K7L), electron density at the interface between the voltage-sensing domain (VSD) and pore domain (PD) was modeled as cholesteryl hemisuccinate (CHS) (Whicher and MacKinnon, 2016). Structural comparison further shows that the residues surrounding this putative sterol-binding site are only partially conserved in Kv11.1 (Figure 4). However, comparable sterol density has not been reported in the other currently available Kv10.1 or Kv11.1 structures. Therefore, whether this represents a physiologically relevant cholesterol-binding site or a structure-specific interaction remains to be established by future structural and functional studies. Supporting the possibility of direct sterol-channel interactions, Varga et al. showed that cholesterol and 7-dehydrocholesterol modulate Kv10.1 through direct targeting of the PD. The inhibition percentage on EAG1 of these two compounds at 195 μM was determined as 28.7% and 41.5%, respectively (Zakany et al., 2019). Together, these findings suggest that sterol regulation of Kv10.1 differs from that of Kv11.1, although the underlying molecular mechanisms remain incompletely understood.

### 4.3 Polyunsaturated fatty acid (PUFA)

Polyunsaturated fatty acids (PUFAs) are important constituents of the plasma membrane, which influence the fluidity, curvature, and ordering of the lipid bilayer (Harayama and Antonny, 2023). Characterized by an anionic carboxylate headgroup and a flexible hydrocarbon tail containing multiple *cis* double bonds, PUFAs are capable of modulating various ion channels, with a particular affinity for voltage-gated potassium channels. For example, Valenzuela et al. demonstrated that arachidonic acid (AA) and docosahexaenoic acid (DHA) significantly inhibit Kv11.1 activity. Specifically, at a concentration of 10 μM, AA and DHA reduced hERG currents by 37.7% ± 2.4% and 50.2% ± 8.1%, respectively. Notably, ETYA (5,6,11,14-eicosatetraynoic acid), a non-metabolizable analog of AA, induced a comparable inhibitory effect. This observation strongly suggests that the modulation of hERG is obtained via direct lipophilic interaction with the fatty acid, rather than through downstream metabolic byproducts. Furthermore, a previous study suggested that PUFAs such as AA and DHA preferentially bind to the open state of the channel (Guizy et al., 2005). While the precise binding interface on hERG remains unclear, comparative studies on Kv7.1 suggest that PUFAs are likely to interact with the voltage-sensing (S4) and pore-gate (S6) segments (Liin et al., 2018).

In contrast to the inhibitory effects observed in Kv7.1 and hERG, AA acts as a potent activator of Kv10.1. Application of 10 μM AA significantly enhanced the hEAG1 current amplitude by 186% ± 21% at +20 mV, and by an even greater 344% ± 66% at −10 mV. Unlike many other voltage-gated channels, hEAG1 is not inhibited but rather activated by AA, with a half-maximal effective concentration (EC_50_) of approximately 4 μM. AA alters channel behavior by shifting the activation threshold in the hyperpolarizing direction, accelerating activation kinetics, and increasing the channel’s open probability. AA with the concentration of 5 μM has also been shown to promote the proliferation and metabolic activity of human melanoma IGR1 cells. This proliferative response is blocked by the selective hEAG1 inhibitor LY97241, suggesting Kv10.1 activation may contribute to AA-induced cell growth (Gavrilova-Ruch et al., 2007).

The potency of PUFA-mediated modulation of Kv10.1 is closely linked to the degree of fatty acid unsaturation. Eicosapentaenoic acid (EPA, 5 double bonds) is demonstrated to be more effective than arachidonic acid (AA, 4 double bonds), which in turn is more potent than linolenic acid (3 double bonds), whereas oleic acid (1 double bond) exhibits little to no activity (Gavrilova-Ruch et al., 2007). Similar to observations in hERG, this modulation appears to arise from direct lipid–channel interactions, as indicated by the activity of the non-metabolizable analog ETYA, although ETYA produces a weaker effect than AA. Mechanistic studies using arachidonoyl-CoA suggest that the relevant interaction site resides in the exoplasmic (outer) leaflet of the membrane since the extracellular application of 10 μM arachidonoyl-CoA reproduces the characteristic voltage shift and accelerated activation kinetics, whereas intracellular application fails to bring out any response (Gavrilova-Ruch et al., 2007). In another study, Brelidze et al. identified undecylenic acid, a fatty acid with a terminal double bond, as a selective EAG1 inhibitor (IC_50_ = 1.1 μM) that binds to the intracellular PAS domain (Wang Z.-J. et al., 2019). This finding highlights the structural specificity underlying lipid regulation, where subtle differences in fatty acid structure can determine whether a molecule functions as an activator or an inhibitor of Kv10.1. Despite these insights, the precise molecular mechanism for the modulation of Kv10.1 (and Kv11.1) by lipid species remains unclear and requires further investigation.

## 5 Future directions and conclusion

As mentioned in the previous sections, the high structural conservation between Kv10.1 and Kv11.1 is the main reason why most existing Kv10.1 inhibitors display strong cross-reactivity with Kv11.1, often binding the off-target channel even more strongly than the intended one. While both channels are strongly regulated by membrane lipids, including PIP_2_, cholesterol, and polyunsaturated fatty acids (PUFAs), despite the similarity in structure, the molecular pathways through which these lipids influence channel gating differ fundamentally. These lipid-dependent regulatory distinctions suggest that channel function may be influenced by the surrounding membrane environment and that this context may contribute to differences between Kv10.1 and Kv11.1 beyond intrinsic structural features. In our opinion, there are two possible strategies for exploiting this divergence.

The first strategy involves the rational design of lipid-mimetic molecules that engage lipid-protein interfaces unique to Kv10.1. The contrasting effects of arachidonic acid (activating Kv10.1 but inhibiting Kv11.1) and the identification of undecylenic acid as a selective Kv10.1 inhibitor provide initial indications supporting this approach. Nevertheless, the precise molecular mechanisms underlying these interactions remain incompletely understood, and further studies are required to define how lipid-channel interactions can be reliably targeted. Additional structural and functional studies are therefore essential to determine how different lipid environments influence Kv10.1 behavior and whether these effects can be exploited in a controlled manner. This is especially important in oncology, where tumor lipid composition is highly dynamic and varies across cancer types, tumor microenvironments, and disease stages. A deeper understanding of which lipid alterations drive Kv10.1 dysregulation at specific stages of tumor progression will be critical for informing precision therapeutic design.

A second promising strategy centers on identifying dynamic allosteric sites, the “hidden pockets” that are transiently accessible only during specific intermediate conformational states. Previous studies revealed that Kv11.1 has three major states (Closed, Activated, and Deactivated), whereas Kv10.1 lacks a stable Deactivated state and instead transitions between Activated and multiple Closed states (Durdagi et al., 2012; Whicher and MacKinnon, 2019). Distinct lipid sensitivities may underlie these divergent conformational landscapes. Even when channels share conserved sequences, the adoption of different intermediate structures suggests that Kv10.1 may possess unique, state-dependent allosteric pockets that are not present or not druggable in Kv11.1. However, applying this strategy to Kv10.1 in the context of cancer may be complicated by the highly dynamic and heterogeneous lipid environment of tumor membranes. Because membrane lipid composition varies substantially across cancer types and even across different stages of disease progression, the regulatory mechanisms and corresponding allosteric landscapes of Kv10.1 are unlikely to be uniform. Progress in this area will therefore depend on consolidating the currently fragmented cancer lipidomics data into comprehensive, high-resolution datasets.

Beyond direct effects on channel gating, alterations in membrane lipid composition may also influence the pharmacological behavior of Kv10.1 in more indirect ways. Changes in membrane order, lipid packing, and microdomain organization can affect drug partitioning, local concentration, and accessibility to binding sites. As a result, disease-associated lipid remodeling may modify not only channel function but also the kinetics and efficacy of drug-channel interactions. Although several studies have tried to investigate drug diffusion across different membrane environments (Zhang et al., 2016; Almeida et al., 2023; Belletto et al., 2026), these aspects remain largely unexplored.

An additional and largely unexplored possibility is that interactions between membrane lipids and Kv10.1 may be bidirectional rather than exclusively driven by membrane lipids. Recent studies have demonstrated that changes in membrane potential can directly modulate the nanoscale organization of anionic phospholipids, particularly phosphatidylserine (PS), within the inner leaflet of the plasma membrane, thereby regulating Ras nanoclustering, ERK signaling, and cell proliferation (Zhou et al., 2015; Sasaki et al., 2025). These findings establish membrane potential itself as an important regulator of membrane lipid organization and downstream signaling pathways. Since Kv10.1 is frequently overexpressed in tumor cells and contributes to the regulation of membrane excitability, it is conceivable that altered channel activity could indirectly influence membrane lipid organization by modifying the membrane potential. If cancer-associated lipid remodeling indeed modulates Kv10.1 function, as proposed throughout this review, altered Kv10.1 activity could in turn influence membrane lipid organization through membrane-potential-dependent mechanisms, thereby establishing a potential positive feedback loop between membrane composition, bioelectric signaling, and channel function. Although direct experimental evidence linking Kv10.1 to voltage-dependent phospholipid reorganization is currently lacking, this hypothesis provides a testable conceptual framework for future studies investigating reciprocal interactions between membrane bioelectricity, lipid organization, and oncogenic signaling.

In conclusion, although Kv10.1 and Kv11.1 share a conserved structural framework, they may be regulated by distinct lipid-dependent regulatory mechanisms. This divergence highlights the importance of considering the membrane environment as an additional layer influencing channel behavior. Future multidisciplinary studies, including molecular dynamics simulations, electrophysiology, structural biology, and lipidomics, will be essential to elucidate how membrane composition shapes Kv10.1 function. More broadly, this perspective suggests that ion channels should be studied within their dynamic membrane context, which may represent an underexplored determinant of both function and pharmacological response.
